# Meta-analysis of STAT3 and phospho-STAT3 expression and survival of patients with breast cancer

**DOI:** 10.18632/oncotarget.23962

**Published:** 2018-01-04

**Authors:** Ya Liu, Jie Huang, Wen Li, Yujuan Chen, Xuejuan Liu, Jing Wang

**Affiliations:** ^1^ Department of Breast Surgery, Western China Hospital of Sichuan University, Chengdu, 610041, China; ^2^ Department of Rheumatology, Shenzhen Hospital of Peking University, Guangzhou Medical University, Shenzhen, 518000, China

**Keywords:** STAT3, p-STAT3, breast cancer, prognostic, meta-analysis

## Abstract

**Objective:**

The prognostic value of signal transducer and activator of transcription 3 (STAT3) and phospho-STAT3 in breast cancer remains controversial in heterogeneous. The objective of this meta-analysis was to evaluate STAT3 and phospho-STAT3 expression on the prognosis of breast cancer patients.

**Materials and Methods:**

PubMed, Cochrane Central Register of Controlled Trials, Embase, Web of Science, Chinese CNKI, and Wan Fang were searched up to 19th June 2017. Studies which investigated the STAT3 or phospho-STAT3 expression of patients with breast cancer on the basis of patient survival data or survival curve were eligible.

**Results:**

This meta-analysis involves 12 studies and 4513 female patients with breast cancer. No clear relationship exists between overall survival (OS) and high expression of STAT3 and p-STAT3 (hazard ratio [HR] = 0.95, 95% confidence interval [CI]: 0.62–1.46, *p* > 0.05). p-STAT3 expression is unrelated to disease-free survival (HR = 0.69, 95% CI: 0.18–2.55, *p* = 0.573). Notably, the pooled effect predicts better breast cancer-specific survival with p-STAT3 overexpression (HR = 0.68, 95% CI: 0.59–0.78, *I*^2^ = 30.9%, *p* < 0.001). Results of subgroup analyses show that STAT3 overexpression indicates shorter OS (HR = 1.87, 95% CI: 1.42–2.45, *p* < 0.001) when excluding the heterogeneity test. Meanwhile, p-STAT3-positive patients have a significantly higher OS than their counterparts (HR = 0.72, 95% CI: 0.57–0.91, *p* < 0.01).

**Conclusions:**

Positive STAT3 expression may indicate poor OS. However, p-STAT3, as a potential molecular biomarker for predicting chemotherapeutic effect, appears to have better prognostic value than STAT3.

## INTRODUCTION

Signal transducer and activator of transcription 3 (STAT3), one of the seven-member STAT family of transcription factors, can be activated by tyrosine kinase signals and dimerized to phospho-STAT3 (p-STAT3) [1]. STAT3 and p-STAT3 are significantly associated with tumor development, migration, and incursion in cancer through angiogenesis and lymph-angiogenesis. STAT3 overexpression indicates poor prognosis in various malignancies, such as hepatic cancer, breast cancer, thyroid cancer, gastric cancer, and melanoma. Consequently, STAT3/p-STAT3 could accurately predict patient prognosis.

To gain insight into the correlation of breast tumor with STAT3/p-STAT3, Li CY et al. (2016) conducted a quantitative meta-analysis of seven studies published up to April 2016. They found that high expression of STAT3 and p-STAT3 predicts poor prognosis in East Asians, while a positive outcome was found in non-East Asians [2]. Chen YJ et al. (2013) simultaneously studied STAT3 and p-STAT3 expression in patients with breast cancer and found that the expression of STAT3 and p-STAT3 is higher in tumor tissue than in normal tissues. However, only the N stage, which positively correlates with STAT3 expression, was identified as the independent predictor of the overall survival (OS) of patients with breast cancer [3]. Meanwhile, Liu X et al. (2014) observed that the disease-free survival (DFS) and 5-year OS of p-STAT3-positive patients is higher than that of their counterparts [4]. Accordingly, we present an updated meta-analysis of the literature on the expression of STAT3 and p-STAT3 in patients with breast cancer.

## MATERIALS AND METHODS

### Search strategy

PubMed, Cochrane Central Register of Controlled Trials, EMBASE, Web of Science, Chinese CNKI, and Wan Fang were searched using the following keywords: “signal transducer and activator of transcription 3” or “STAT3”; “phosphorylated signal transducer and activator of transcription 3” or “p-STAT3” or “phospho-STAT3,” and “breast cancer” or “breast carcinoma” or “breast neoplasms” or “mammary cancer” up to 19th June 2017.

### Study eligibility criteria

Included studies were those that (1) recruited patients with the histologic type of breast cancer; (2) measured the expression of STAT3 and p-STAT3 in tumor tissue by immunohistochemistry (IHC); (3) provided data concerning the correlation between STAT3/p-STAT3 expression and survival outcome or clinicopathological information, including tumor differentiation, TNM stages, lymph node metastasis, hazard ratio (HR), and their 95% confidence intervals (CI) or sufficient survival data; (4) used overlapping samples, from which the most complete ones were selected.

Exclusion criteria were (1) duplicate reports, ongoing studies, literature published as abstracts, letters, editorials, theoretical papers, reviews, and case reports; (2) studies on breast cancer cell lines and animal models but not based on human patients; (3) studies that assessed the effect of medication without reporting STAT3/p-STAT3 expression and breast cancer; (4) studies without sufficient survival data to obtain HR and 95% CI; (5) studies whose research methods and experimental design were apparently dissimilar from those of the selected studies.

### Data extraction

Two investigators (LY, HJ) independently examined all literature searches to recognize eligible studies, abstracted data from all studies, and crosschecked data. Controversial problems were resolved by discussions between the two examiners. For each included study, we recorded baseline characteristics, histology, stage, grade, estrogen receptor (ER) and progesterone receptor (PR), human epidermal growth factor receptor 2 (HER2) status, cut-off value of positive expression, antibodies used for IHC, survival curves, and HRs and their 95% CIs and OS, DFS, or cancer-specific survival (CSS) rate. If the only obtainable data of HR and 95% CI were in the form of the survival curve, then Engauge Digitizer version 4.1 (downloaded from http://digitizer.sourceforge.net/) was used to read the graphical survival plot [5]. The Newcastle–Ottawa Scale criterion was used by the two reviewers to independently assess the quality of each included study [6]. Newcastle–Ottawa Scale mainly includes subject selection, subject comparability, and ascertainment of exposure risk. NOS scores ranged from 0 to 9, and a score ≥ 7 indicates as high-quality studies.

### Statistical analysis

The time-to-event HR estimates were pooled into a summary HR by meta-analysis. Heterogeneity test of the individuals was evaluated with Cochrane *Q* test and the I^2^ statistic. Heterogeneity was presented as significant when I^2^ > 50% or *p* value of *Q* test < 0.1, and random-effects model was adopted if there was significant heterogeneity, otherwise, the fixed-effects model was adopted [7]. Galbraith plot was worked for the heterogeneity test. Furthermore, publication bias was measured using Egger’s linear regression test [8]. All statistical calculations were performed via Stata version 12.0 (Stata Corp, College Station, TX, USA). *P* < 0.05 was considered statistically significant [9–11].

## RESULTS

### Search results

**Figure 1:**
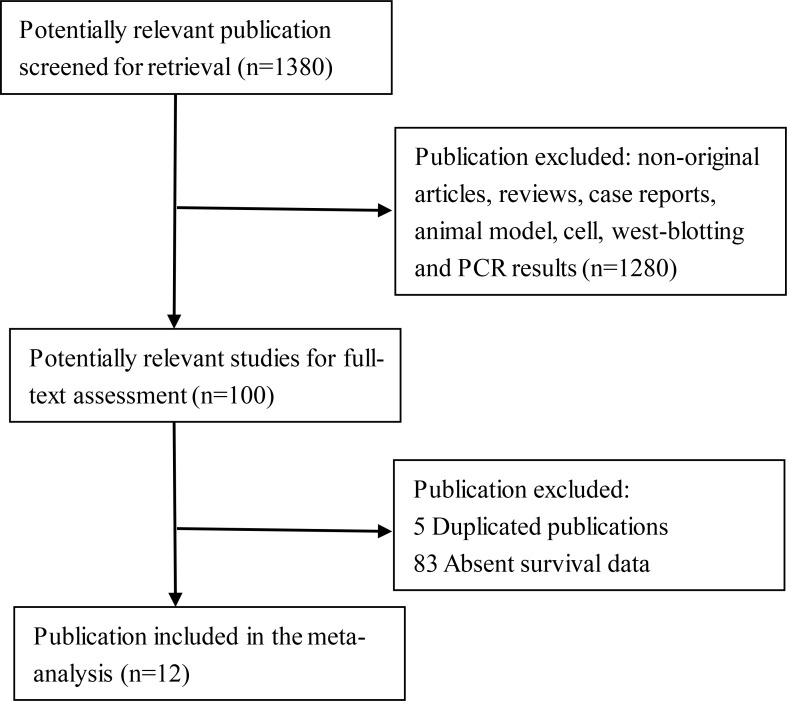
Flow diagram of reviewed relevant publications

The search strategy identified 1380 studies. Details of the flow diagram of the identification and attrition of studies are shown in Figure 1. In accordance with the inclusion criteria, 12 studies with full text (Dolled F. et al. 2003; Yamashita H. et al. 2006; Liu C. et al. 2007; Sheen C. et al. 2008; Li SJ. et al. 2011; Sato T et al. 2011; Sonnenblick A. et al. 2013; Chen YJ. et al. 2013; Liu X. et al. 2014; Zhang N. et al. 2016; Aleskandarany MA et al. 2016; Fadia JA. et al. 2016) were included, with 4513 female breast cancer patients who underwent surgery. STAT3 and p-STAT3 expression was evaluated by IHC in all studies. Further details on the included studies are listed in Table 1 [3, 4, 12–21].

**Table 1:** Main characteristics and results of eligible studies

Studies	Patient source	Histology	N pts	STAT3+ (%)	p-STAT3+ (%)	Age (years)	Clinicopathologic features	Method Antibody	Cut-off value	IHC Score	Follow up (months)	Survival analysis	HR	95% CI	HR statistics	Quality score
Dolled F. (2003)	USA	Node-negative	346	C198 (69.2); N 66 (23.1)	C 56 (19.6); N124 (43.5)	NR	ER, PR, Her2, Ki-67, T, LN, G	IHC/ NR	Score=1	a	187	OS	0.35 0.43^P^	0.12-1.03 0.18-0.99^P^	R	6
Yamashita H. (2006)	Japan	Invasive ductal cancer	517	213 (41.2)	NR	22–91	ER, PR, Her2, T, G, LN	IHC/m	10%	b	97	OS/DFS	1.28 0.82^#^	0.84-1.95 0.59-1.14^#^	SC	8
Liu C. (2007)	China	Unselected	130	NR	83 (63.8)	41–68 (55 median)	ER, PR, Her2, Ki-67, T, G, LN	IHC/ NR	Score=3	c	61	OS/DFS	1.12^P^ 1.30^P#^	0.58-2.16^P^ 0.64-2.63^P#^	R	7
Sheen C. (2008)	China	Invasive breast cancer	102	27 (26.5)	NR	48.2	ER, S, T, G, LN	IHC/r	Score=3	a	69.7	OS	2.35	1.40-3.04	R	8
Li SJ. (2011)	China	Invasive ductal cancer	67	42 (62.7)	NR	32–67 (50.7 median)	S, T, LN	IHC/ r	Score=3	c	70.6	OS	3.88	1.12-13.43	R	8
Sato T. (2011)	USA	Unselected	721	NR	371 (51.5)	30–98 (64 median)	T, G, LN	IHC/ r	Score=1	c	150	OS	0.84^P^	0.62-1.12^P^	R	7
Amir S. (2013)	Israel	Invasive ductal cancer	375	NR	134 (35.7)	50 (median)	ER, PR, Her2, T, G, LN	IHC/ r	Score=1	c	108	OS	0.48^P^	0.28-0.84^P^	R	7
Chen YJ. (2013)	China	Invasive ductal cancer	140	87 (62.1)	67 (47.9)	32–77 (48.8 median)	ER, PR, Her2. Ki-67, S, T, G, LN	IHC/ r	Score=1	c	54	OS	2.38 1.65^P^	0.60-9.35 0.57-4.76^P^	SC	7
Liu X. (2014)	China	Invasive ductal cancer	82	NR	31 (37.8)	30–73 (52 median)	ER, PR, Her2, T, G, LN	IHC/ r	Score=1	c	60	OS/DFS	0.30^P^ 0.34^P#^	0.09-1.03^P^ 0.14-0.85^P#^	R	7
Zhang N. (2014)	China	Unselected	379	NR	146 (38.5)	NR	ER, PR, Her2, S, T, LN	IHC/ r	Score =2	c	166	OS	1.14^P^	0.36-3.67^P^	R	8
Aleskandarany M. (2016)	UK	Unselected	1270	NR	675 (53.1)	NR	ER, PR, Her2, Ki-67, T, G, LN	IHC/ NR	30	c	NR	CSS	0.77^**^	0.60-0.99^**^	R	6
Fadia JA. (2016)	UK	Invasive ductal cancer	384	NR	230 (59.9)	NR	ER, PR, Her2, T, G, LN	IHC/ r	M	c	148	CSS	0.64^**^	0.64-0.90^**^	R	6

Table 1.N pts, number of patients; NR, not reported; R, reported; HER2, human epidermal growth factor receptor 2; ER, estrogen receptor; PR, progesterone receptor; S, stage; G, grade; T, tumor size; LN, lymph node status; IHC, immunohistochemistry; M, moderate; r, rabbit; m, mouse; IHC score, a, Staining intensity; b, Percent of positive cells; c, Percent of positive cells multiply staining intensity equals to final product score; SC, survival curve; OS, overall survival; DFS, disease-free survival; CSS, cancer-specific survival; HR, hazard ratio; 95% CI, 95% confidence interval. ^p^, phospho-STAT3; ^**^, HR and 95% CI of CSS; ^#^, HR and 95% CI of DFS.

### Meta-analysis

In all selected studies, five publications analyzed the prognostic value of STAT3 expression, nine of p-STAT3 expression, and two of STAT3 and p-STAT3 expression. In addition, 10 cohorts investigated the STAT3 and p-STAT3 expression and OS of patients, 3 studies examined DFS, and 2 studies determined breast CSS (Table 1).

**Figure 2:**
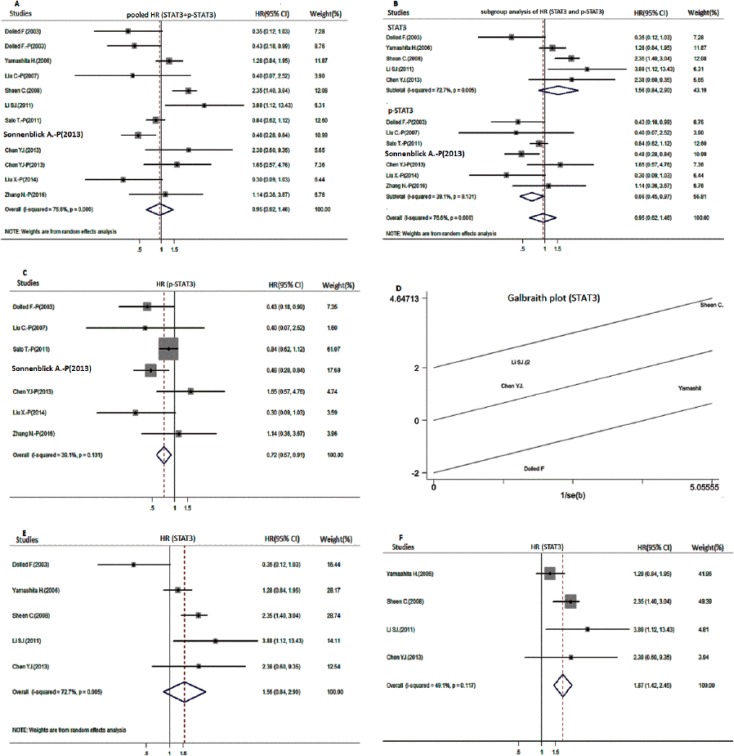
Meta-analysis for the overall survival of positive STAT3 and p-STAT3 expression in patients with breast cancer (**A**) Forest plot of pooled hazard ratios (HR) for the pool overall survival (OS); (**B**) HR for different biomarker subgroup analysis; (**C**) HR for the OS of positive p-STAT3 expression in the fixed-effects model; (**D**) Galbraith plot for the heterogeneity test of positive STAT3 expression; (E and F) HR for the OS of positive STAT3 expression before (**E**) and after (**F**) excluding the results of the heterogeneity test.

Considering the significant heterogeneity (*I*^2^ = 76.6%, *p* value of *Q* test for heterogeneity test < 0.001), we adopted the random-effects model to estimate the pooled outcome. The pooled HR and 95% CI of the OS for all 10 studies showed no clear relationship to STAT3 and p-STAT3 overexpression (HR = 0.95, 95% CI: 0.62–1.46, *p* = 0.808) (Figure 2A). Meanwhile, meta-analysis adopting the random-effects model revealed that p-STAT3 expression is unrelated to DFS (HR = 0.69, 95% CI: 0.18–2.55, *p* = 0.573). Interestingly, pSTAT3-positive patients indicate better breast cancer-specific survival than pSTAT3-negative patients in the random-effects model (HR = 0.68, 95% CI: 0.59–0.78, *I*^2^ = 30.9%, *p* < 0.001).

Subgroup analyses of HR for the OS of STAT3 and p-STAT3 expression were performed. First, subgroup analysis was performed to estimate whether biomarkers (STAT3 and p-STAT3) of breast cancer are different from the analysis above (Figure 2B). Results showed that p-STAT3-positive patients have significantly higher OS than their counterparts (combined HR = 0.66, 95% CI: 0.45–0.97) in the random-effects model. Considering that *I*^2^ = 39.1% and *p* value of *Q* test for heterogeneity test = 0.131, we tested p-STAT3 again in the fixed-effects model (HR = 0.72, 95% CI: 0.57–0.91) (Figure 2C). However, another outcome displayed a significant degree of heterogeneity in the STAT3 subgroup (*I*^2^ = 72.7%, *p* value of *Q* test for heterogeneity test < 0.1). Accordingly, we performed heterogeneity test and sensitivity analysis. Dolled F. (2003) performed a heterogeneity test by applying the Galbraith plot (Figure 2D). Subsequently, we calculated pooled HRs again for the OS of STAT3 before and after excluding the heterogeneity test. Results showed that STAT3 overexpression reduces OS (HR = 1.87, 95% CI: 1.42–2.45, *p* < 0.001) when the result of the heterogeneity test is excluded (HR = 1.56, 95% CI: 0.84–2.90, *p* = 0.163) (Table 2, Figure 2E and 2F). In all outcomes mentioned above, a difference between STAT3 and p-STAT3 expression for OS in breast cancer was observed. We also performed subgroup analysis according to race (Asian and non-Asian), year (published before and after 2010), patient number (over and under 100 patients), and IHC scoring method (multiplied score and staining intensity or percent of positive cells) (Table 3). Overall, no significant association was observed. However, the subgroup analysis of non-Asian participants (0.57, 95% CI: 0.37–0.88, *I*^2^ = 50.7%, *p* = 0.012) predicted good OS.

**Table 2:** Test of heterogeneity and sensitivity analysis in the STAT3 subgroup

Sensitivity analysis	Heterogeneity		Pooled effect	
	I^2^	*P* value of *Q* test	HR and 95% CI	*P* value
Include Dolled F. (2003)	72.7%	0.005	1.56 (0.84–2.90)^a^	0.163
Exclude Dolled F. (2003)	**49.10%**	**0.117**	**1.87 (1.42–2.45)^b^**	**0.000**

Table 2.^a^, random-effects model; ^b^, fixed-effects model.

**Table 3:** HR value of the overall survival in breast cancer subgroups according to year, N pts, methods of detecting STAT3 expression, biomarker, race

Stratification	Pooled HR (95% CI) Random effects	I^2^(%)	*p* value of *Q* test
Year			
≤ 2010	0.85 (0.41–1.78)	82.3	0.000
> 2010	0.98 (0.58–1.65)	63.8	0.011
N pts			
≥ 100	0.94 (0.60–1.45)	76.8	0.000
< 100	1.08 (0.09–13.22)	88.0	0.004
IHC scoring method			
Multiply score	0.93 (0.56–1.52)	59.2	0.016
Non-multiply score	0.92 (0.42–2.02)	85.6	0.000
Biomarker			
STAT3	**1.87 (1.42–2.45)**	**49.1**	**0.117**
P-STAT3	**0.72 (0.57–0.91)**	**39.1**	**0.131**
Race			
non-Asian	**0.57 (0.37–0.88)**	**50.7**	**0.108**
Asian	1.42 (0.88–2.31)	58.5	0.018

Table 3. N pts, number of patients; IHC, immunohistochemistry; HR, hazard ratio; 95% CI, 95% confidence interval.

The *p* value of Egger’s test (*p* = 0.791) did not indicate publication bias.

## DISCUSSION

STAT3 is a vital cytoplasmic transcription factor, and activated STAT3 (p-STAT3) is upregulated by aberrant upstream tyrosine kinases; p-STAT3 transports information to the nucleus and controls gene expression, which is essential for cancer cell growth and survival [1]. Xu YH et al. (2014) performed a meta-analysis of 17 retrospective trials, including 1793 patients with non-small-cell lung cancer. They found that positive STAT3 expression in patients is associated with poorly differentiated, advanced stage, lymph node metastasis. They also revealed that the estimated pooled HR (0.67, 95% CI: 0.57–0.77) is statistically significant for the OS of patients with non-small-cell lung cancer (*p* < 0.0001) [22]. Li MX et al. (2015) performed a meta-analysis of the digestive system by enrolling 22 studies with 3585 patients. Similarly, they found that p-STAT3 overexpression is related to tumor differentiation and lymph node metastases. On the relationship between breast tumor and STAT3/p-STAT3 expression, the overexpression of STAT3 and p-STAT3 also exerts a similar effect on non-small-cell lung cancer and digestive system cancer in poorly differentiated, advanced stage, lymph node metastasis [23]. Considering that the estimated pooled HR (0.90, 95% CI: 0.63–1.28, *p* = 0.557) is unrelated to OS, Li CY et al. (2016) conducted a subgroup analysis and found that the pooled HRs of OS differ with the origin change of population [2]. Consequently, we performed the present meta-analysis to determine whether the combined effect of HR between positive STAT3/pSTAT3 expression and OS is similar.

This meta-analysis involves 12 studies and 4513 female patients with breast cancer. This study found no clear relationship between OS and high STAT3 and p-STAT3 expression (HR = 0.95, 95% CI: 0.62–1.46, *p* > 0.05). Thus, we reviewed the enrolled literature searches again and found that both Sheen C. (2008) and Li SJ. (2011) reported that STAT3 is related to poor clinicopathological parameters and poor OS [15, 16]. However, both Sonnenblick A. (2013) and Liu X. (2014) testified that p-STAT3 is a marker of good prognosis in breast cancer [4, 18]. Therefore, we analyzed whether the pooled insignificant HR is interconnected with different biomarkers (STAT3 and p-STAT3) of breast cancer. We performed subgroup analysis and found that STAT3 overexpression reduces OS (HR = 1.87, 95% CI: 1.42–2.45, *I*^2^ = 49.10%, *p* < 0.001) in the fixed-effects model when the results of the heterogeneity test involving patients with node-negative breast cancer are excluded. Chen YJ. et al. (2013) reported that positive p-STAT3 expression possibly indicates poor OS in breast cancer because it is significantly associated with tumor formation, migration, and invasion. By contrast, our meta-analysis found that pooled positive p-STAT3 expression indicates significantly better OS (HR = 0.72, 95% CI = 0.57–0.91, *I*^2^ = 39.1%, *p* = 0.006) in the fixed-effects model. Prior studies on other types of cancer have reported a similar observation. Monnien F et al. (2010) detected the nuclear expression of p-STAT3 in 104 cases of advanced rectal cancer (T3–T4) by IHC [24]. They argued that the OS of p-STAT3-positive patients is significantly higher than that of their counterparts. However, the underlying mechanisms remain unclear. A possible explanation is that p-STAT3-positive patients may be associated with better response to neoadjuvant and adjuvant chemotherapy because activated p-STAT3 could contribute to microtubule formation and promote tumor cell dissemination. Meanwhile, neoadjuvant or adjuvant chemotherapy, such as Paclitaxel, possibly inhibits microtubule formation and transactivation activity. By contrast, neoadjuvant therapy may inhibit STAT3 phosphorylation and may negatively impact dimerization. Yuan B et al. (2008) detected the nuclear expression of p-STAT3 before and after neoadjuvant therapy in 50 cases of cervical cancer [25]. They found that the proportion of genes in high expression level of p-STAT3 decreased from 92% to 76% (*p* = 0.001). Similarly, Sonnenblick A. et al. (2013) claims that high p-STAT3 expression is connected with improved OS only among breast cancer patients who received adjuvant chemotherapy [18]. These results indicate that p-STAT3 is a potential molecular biomarker for predicting chemotherapeutic effect, and patients with positive p-STAT3 could easily benefit from chemotherapy. Furthermore, both targeting STAT3 for blocking either dimerization or STAT3 phosphorylation and targeting p-STAT3 can directly improve prognosis. This condition may also be the reason why the pooled effect predicts better breast CSS with p-STAT3 overexpression (*p* < 0.001). Nevertheless, trials associated with breast cancer CSS and DFS were relatively rare. We expect an updated pooled effect about both CSS and DFS through further validation using a large cohort of patients and prospective randomized data in breast cancer.

Additionally, STAT3 is phosphorylated by various upstream genes. Tell RW et al. (2014) demonstrated that p70S6K phosphorylation and JNK signaling could induce pSTAT3 formation in basal-like cancers, SRC Y527 and EGFR Y1068 phosphorylation prompt STAT3 phosphorylation in luminal A, and PKC α S657 and YB-1 S102 stimulate STAT3 phosphorylation in luminal B-type breast cancers [26]. Meanwhile, Li GC et al. (2014) revealed that continuing STAT3 signal mediates trastuzumab resistance through the overexpression of MUC1 and MUC4 in primary HER2-positive breast cancer. Then, silencing both MUC1 and MUC4 could mostly recover trastuzumab sensitivity [27]. Different upstream gene expressions related to STAT3 signaling have different impacts on human breast cancer subtype [28]. Thus, we hypothesize that various upstream signaling pathways of STAT3 contribute to distinct prognosis in different breast cancer subtypes. Unfortunately, limited research focused on the prognostic role of STAT3 tumor cell expression in different molecular subtypes. Therefore, additional evidence is necessary to verify the association between STAT3 activation and OS in different breast cancer subtypes.

Although the present meta-analysis suggests the absence of publication bias (*p* > 0.1), we could not prevent potential bias among these studies. Despite our significant efforts to gather additional relevant information, publication bias was still inevitable. Small-sample or negative studies are unlikely to be accepted and published. Furthermore, some reports did not offer sufficient data for HRs and 95% CIs. We obtained data by reading the survival rates presented on the graphical survival plot. Nevertheless, this method cannot completely eliminate inaccuracy. Authentic conclusions cannot be drawn in the absence of all relevant information. Thus, large-scale RCT studies with long-term follow up and a full description of survival events are indispensable.

In conclusion, positive STAT3 expression may indicate poor OS. However, p-STAT3, as a potential molecular biomarker for predicting chemotherapeutic effect, appears to have better prognostic value than STAT3.

## CONCLUSIONS

Positive STAT3 expression may indicate poor OS. However, p-STAT3, as a potential molecular biomarker for predicting chemotherapeutic effect, appears to have better prognostic value than STAT3.

## Abbreviations

STAT3: signal transducer and activator of transcription 3
p-STAT3: phospho-STAT3
OS: overall survival
HR: hazard ratio
CI: confidence interval
DFS: disease-free survival
CSS: cancer-specific survival
IHC: immunohistochemistry
ER: estrogen receptor
PR: progesterone receptor
HER2: human epidermal growth factor receptor 2
